# Diphenoxylate-atropine (Lomotil) Toxicity in Infantile Diarrhea: A Case Report of Therapeutic Failure

**DOI:** 10.7759/cureus.5875

**Published:** 2019-10-09

**Authors:** Hamza R Khan, Sarrah Ali Asghar, Sharfa Kanwal, Laila Tul Qadar, Kashif H Qadri

**Affiliations:** 1 Internal Medicine, Quaid-e-Azam Medical College, Bahawalpur, PAK; 2 Internal Medicine, Dow University of Health Sciences, Karachi, PAK; 3 Pediatrics, Dow University of Health Sciences, Karachi, PAK

**Keywords:** diphenoxylate-atropine toxicity, drug contraindications, diarrhea, dosage, naloxone, lomotil

## Abstract

Diphenoxylate-atropine (Lomotil) intoxication incidence was significantly high in the past, but seeing such cases in the present day of modern and advanced medicine, hints about the gaps in the practice of medicine. In our case, a general physician maltreated an infant for diarrhea with an adult dose of diphenoxylate-atropine (Lomotil), a Food and Drug Administration (FDA) unapproved drug, which caused labored breathing and pinpoint pupils. After being maltreated, at the time of presentation to the emergency room (ER), she was being misdiagnosed as a case of dehydration until doctors noticed miosis and reached the diagnosis of diphenoxylate-atropine (Lomotil) toxicity. Her condition completely reversed with a single dose of naloxone. Hence, this case highlights the need for basic knowledge about the dosage of drugs for different age groups, especially infants, along with the importance of adherence to the evaluation protocols for accurate management.

## Introduction

Diarrhea is a dangerous sign for the health of a child. Hence, it requires appropriate and timely management. The first-line approach for managing a case of diarrhea is physiological, which includes rehydration, either orally or through the intravenous route. The pharmacological method comprising antidiarrheals as adjuvant therapy is also used [1]. The most common antidiarrheals prescribed by general physicians in our country are diphenoxylate-atropine (Lomotil) and loperamide [2].

Lomotil is a synthetic substance that is chemically related to meperidine. A subtherapeutic dosage of atropine, an anticholinergic, is added to the preparation, to discourage deliberate overdose. It has the role of an antisecretory agent when it acts on enteric μ-opioid receptors, resulting in the release of noradrenaline as a final mediator. It also acts as a spasmogenic to prolong gastric emptying and decrease bowel frequency, thus rendering the gut atonic [3-4]. The use of diphenoxylate-atropine (Lomotil) in higher than the prescribed dosage can cause toxicity. Clinical presentations in a case of toxicity vary in terms of which a component of the drug (opioid or anticholinergic) will dominate first. The metabolism of diphenoxylate involves rapid and extensive conversion by ester hydrolysis into difenoxin, which is biologically five times more active and a major metabolite in the blood [5]. Antidiarrheal diphenoxylate-atropine (Lomotil) is available in forms like tablets and syrup. The composition of a single pill of diphenoxylate-atropine (Lomotil) is 2.5 mg of diphenoxylate and 0.025 mg of atropine sulfate. The dose of usually two tablets, which is prescribed to adults only produces required opioid effects locally in the gut to control diarrhea. However, the same treatment can cause central nervous system (CNS) and respiratory depression in an infant or child.

Despite well-documented risks and toxicities of this drug as well as contraindications of its use at an age of fewer than two years by the Food and Drug Administration (FDA) [6], doctors of our locality still prescribe it with no important instructions to parents. Herein, we present a case of diphenoxylate-atropine (Lomotil) toxicity in a 10-month-old infant treated for diarrhea.

## Case presentation

A 10-month-old female child presented in the emergency room (ER) of Dr. Ruth KM Pfau, Civil Hospital Karachi (CHK), with an altered level of consciousness and shallow breathing. The child was vaccinated according to the expanded program on immunization (EPI) but was malnourished, with an unsubstantial history of family illnesses. The patient was accompanied by her mother. She weighed 6.6 kg, is the fourth-born child to her parents and was delivered at term to a 37-year-old G4P4 (gravida 4 para 4) mother via normal vaginal delivery. The mother did not disclose any complications during pregnancy. 

The patient developed respiratory distress since that morning accompanied by low-grade intermittent fever on the day of presentation. She had a history of loose, watery diarrhea for one day. There were 12 episodes of watery motions, not blood-stained, for which treatment was taken by the nearby general physician, including antibiotics, zinc supplements, and two tablets of diphenoxylate-atropine after which diarrhea was resolved. Past medical history revealed the child is developmentally delayed and is being treated for maculopapular rash. The mother also noticed weight loss and altered bowel habits, although micturition was normal.

On examination, the patient was lying on the bed, irritated and lethargic, having labored breathing. Initial vitals included blood pressure (BP) 129/103 mmHg, a regular pulse of 201 beats/min, a respiratory rate of 15 breaths/min, and a low-grade fever of 99.7° F. The patient was anemic and dehydrated, with no visible signs of clubbing, cyanosis, edema, and lymphadenopathy. Various lab investigations were ordered, which include complete blood count (CBC) (Table 1), urea creatinine electrolytes (UCE) (Table 2), and arterial blood gas (ABG) (Table 3). All other systems were unremarkable.

**Table 1:** CBC of our patient CBC: Complete blood count

Parameters	Result	Normal Value
Hemoglobin (g/dL)	12.3	11-16
Mean corpuscular volume (fl)	91.6	82-95
Mean corpuscular hemoglobin (pg)	32.2	27-31
Mean corpuscular hemoglobin concentration (g/dL)	35.1	32-36
Total leukocyte count (10^9^/L)	10.7	4-11
Neutrophils (%)	32.7	40-70
Lymphocytes (%)	46.7	20-45
Platelets (10^3^/μl)	370	150-450

**Table 2:** Values of UCE in our patient UCE: Urea creatinine electrolytes

Parameters	Result	Normal Value
Blood urea nitrogen (mg/dL)	6	7-20
Creatinine (mmol/L)	0.3	0.6-0.12
Na (mmol/L)	137	135-145
K (mmol/L)	4.6	3.5-5.1
Cl (mmol/L)	103	98-107

**Table 3:** An ABG test values of our patient ABG: Arterial blood gas

Parameters	Result	Normal Value
pH	7.35	7.35-7.45
pCO_2 _(mmHg)	48	35-45
HCO_3_^-^ (mmol/L)	20	22-26

On chest auscultation, harsh vesicular breathing and equal air entry were heard; the breathing pattern was abnormal and shallow. The presumptive diagnosis of dehydration was made. However, when reflexes were found to be brisk, with a pinpoint pupil, the diagnosis of opioid (diphenoxylate-atropine) toxicity was made, which was completely reversed by a single naloxone dose of 0.6 mg. The child responded immediately after the administration of naloxone and the breathing pattern also improved. The pinpointed pupil got settled toward dilation and became reactive. The level of consciousness was also regained.

## Discussion

Diphenoxylate-atropine (Lomotil) ranks seventh on the list of deadly drugs that cause severe intoxication in children with just a single dose [7]. Anticholinergic overdose symptoms, from the most to the least common, include tachycardia, restlessness/anxiety, flushing, urinary retention, and diminished reflexes, while overdose symptoms due to the diphenoxylate component of diphenoxylate-atropine (Lomotil), from the most to the least common, are drowsiness, vomiting, respiratory depression, coma, and abdominal pain/constipation [7]. Miosis, seizures, or paralytic ileus may also be seen. Our patient exhibited many symptoms; however, the only point of diagnosis was the presence of miosis whereas other reports show that ocular toxicity, ototoxicity, and cardiac conduction defects like arrhythmias can occur [8].

Our infant patient was also given a single adult dose of two tablets for the treatment of diarrhea, which was controlled at the time of presentation to the ER. Presenting complaints were delayed sensorium, altered level of consciousness, and other signs and symptoms (S/S) mentioned above. Initially, the doctor thought that the patient is a simple case of dehydration, so she was being managed for it until her pupils were found to be pinpoint, which, on further inquiry from the mother, shifted the diagnosis to diphenoxylate-atropine (Lomotil) toxicity.

As per reports, there are usually two phases of diphenoxylate-atropine (Lomotil) intoxication [5]. The first phase, which attributed to the dominant effects of atropine, shows S/S such as flushing, high fever, and tachypnea followed by the second phase of opioid dominance due to diphenoxylate mainly comprises CNS and respiratory depression along with miosis. Although our patient presented after almost 24 hours of taking diphenoxylate-atropine (Lomotil) when the phase of atropine was over, she still showed overlapping atropine effects, such as fever and severe tachycardia, co-occurring with bradypnea and miosis, which is an opioid overdose symptom. Moreover, reflexes are diminished as part of atropine effects, but our patient had normal muscle tone and brisk reflexes.

Medical complications seen in this type of toxicity are aspiration pneumonia, cortical blindness, and cerebral edema [5]. In the early stage of diphenoxylate poisoning in children, there is a report of multiple and extensive edema-necrosis in various areas of the brain observed through magnetic resonance imaging (MRI), which also supports the risk of complications [9]. This patient, however, did not suffer any complications.

Various modalities of treatment were adopted in the past like emetics, gastric lavage, activated charcoal, forced diuresis, and, finally, the drug of choice, naloxone [5]. Whereas, in cases of the dependence of diphenoxylate combination therapy of buprenorphine 2 mg and naloxone 0.5 mg is also documented [10]. Our patient’s condition was reversed by just one dose of naloxone.

Toxicity reports for this drug fall under both accidental ingestion and therapeutic administration. The risk of accidental ingestion remains whereas intoxication due to therapeutic administration is not frequently encountered due to education and awareness among doctors. However, to our dismay, cases of toxicity are still seen due to malpractice of some primary care providers mainly to satisfy distressed parents. However, the lack of proper education of parents by the physician regarding dosage and intoxication can sometimes lead to instances where parents overdose the children due to a lack of knowledge about drugs [6]. In our case, the infant was maltreated by the general practitioner of the locality.

Therefore, we find it necessary to report this to educate general practitioners and improve the careful and appropriate management of common pediatric illnesses. We also emphasize the lesson that proper and thorough physical evaluation must be carried out before reaching any diagnosis and starting any treatment. If this infant had been misdiagnosed initially as a case of severe dehydration at the time of presentation to the ER because of missing an essential step of evaluating the pupils, her condition would have remained the same or deteriorated, and she might have died due to intoxication. Hence, this case is different, after considering the therapeutic errors in drugs of known toxicity due to a lack of awareness among general practitioners regarding pediatric dosage and contraindications, evaluation protocols, and approach to management.

## Conclusions

Up-to-date information about the new drugs for common illnesses, along with knowledge of known toxicities of conventional drugs, must be known to the doctors. Apart from that, the dosage of drugs must always be correctly determined and prescribed to the pediatric age group since they are very susceptible to dosage intoxication. For this, all doctors should regularly check FDA guidelines for the drugs that are commonly used for treating such diseases. Moreover, ER care doctors must strictly adhere to evaluation protocols and thoroughly examine patients before deciding because they have a narrow margin of error.
